# Cases from the Surgical Clinic of the Jefferson Medical College

**Published:** 1860-03

**Authors:** Emil Fischer

**Affiliations:** Philadelphia


### ART. I.—*Cases from the Surgical Clinic of the Philadelphia Hospital.* Service of Professor GROSS.

Reported by EMIL FISCHER, M.D., of Philadelphia.

I. *Case of Large Tumor of the Left Labium; Excision.*—Susan P., a colored woman, twenty-six years of age, married, was admitted into the surgical wards of the Philadelphia Hospital, in January, 1860, on account of a large tumor of the left labium. The swelling was noticed by the patient for the first time about two years and a half ago, when it had attained the size of a walnut. Since then it had undergone a steady and gradual enlargement, growing as it seemed at first in length, but increasing of late principally in circumference. On examination the tumor was found to be nearly of the size of an infant's head, of an ovoidal form, larger below than above; it occupied the left labium descending from between the vagina and the tuberosity of the ischium nearly to the middle of the left thigh; it did not seem to have any connection with the pelvis, and felt like a smooth, firm, and elastic body. The skin covering it was perfectly normal. Pressure of the tumor produced some uneasy sensation in it, but otherwise the patient did not complain of any pain, and was merely annoyed by a feeling of weight and tension in the affected parts. Her health had always been excellent.

The tumor, which was evidently of a non-malignant character, was assumed to be of the fibrous kind. Extirpation having been decided upon, the patient was put under preparatory treatment, consisting of rest, diet, and a dose of purgative medicine.

On the twenty-fifth of January, Professor Gross proceeded to remove the tumor before his class. The patient being under the influence of chloroform, and all hair having been shaved off the labium, she was put in the position for lithotomy. Supporting the tumor with his left hand, Professor Gross made two incisions about five inches in length on the inside and on the outside of it, including an elliptical piece of skin, the widest part of which was at the bottom of the tumor, and separating it from the surrounding cellular tissue, partly by careful dissection, partly by using the handle of the scalpel and the finger, removed it completely. The hemorrhage was but slight, and was easily arrested, no ligatures being required. After the bleeding had completely ceased, the margins of the wound were approximated with silver sutures, with the exception of the lower part, into which a tent was inserted.

The woman was ordered to take a dose of morphia, and the cold-water dressing was applied to the wound. On the second day, the patient suffering from some fever, the saline and antimonial mixture was prescribed. Some days afterwards suppuration having been established, and the discharge becoming rather offensive, the parts were dressed with Labarraque's solution. The fever following the operation was but slight. No bad symptoms occurred, and the wound is now healing well and rapidly under the use of appropriate applications.

*Examination of the tumor.*—The tumor weighed about three pounds and a half, and consisted of a large fleshy mass with a somewhat elastic feel, and a smooth and even surface. On making a section of it, a clear limpid fluid oozed out, which coagulated spontaneously. It was composed principally of a succulent, yellow substance, resembling fat, and intersected with opake white bands. Its infiltrated condition gave it very much the appearance of œdematous cellular tissue. Professor Gross pronounced it to be a cellulo-fibrous tumor, an opinion which was corroborated by microscopic examination.

*Remarks.*—The parenchyma of the labia, said Professor Gross, is liable to become the seat of various new formations, such as encysted, fibrous and cellulo-fibrous tumors, scirrhus, and encephaloid.

*Fibrous tumors* do not always originate in the vulva, but may commence to grow from a point deep in the pelvis, appearing at the vulva, only after having made their way from a considerable distance. If they are developed in the substance of the labia, they may attain a considerable volume, owing to the elasticity and dilatability of the tissues of these parts; they may distend the labium enormously, elongate it, and drag it downward to a greater or less extent. They are generally free from pain, and become troublesome only by their weight and size. The skin covering them is, in most cases, perfectly normal. If the tumor is, however, very heavy and voluminous, it may be accompanied by excoriation and ulceration of the skin of the labium. In cases of this kind, the tumor itself may become the seat of inflammatory action, and, undergoing softening and ulceration, may give rise to an abundant discharge of pus. Fibrous tumors of the labia, like those of the uterus, contain not unfrequently small cavities, resembling cysts, which are filled with a gelatinous, sanguinolent, or a clear, serous fluid, or with earthy matter. This peculiarity of structure is met with particularly in tumors of large size, and seems to be the cause of their rapid growth.

The *cellulo-fibrous* tumors, of which the preceding case affords an example, are considered by some pathologists as a softer and more elastic form of the fibrous tumor. They consist essentially of fibro-cellular tissue, infiltrated with a serous, or more or less viscid fluid. Cases of removal of immense cellulo-fibrous tumors have been recorded by Messrs. Lawrence, Liston, and others.

Upon the origin of fibrous tumors, and their mode of development, pathology has as yet thrown but little light; some authors believe that they take their rise in small effusions of blood which have not been completely absorbed. Professor Gross regards them as local hypertrophies, or enlargements, produced by inflammatory action. Experience shows that they are frequently developed during pregnancy, and that this state exercises a marked influence upon their growth; the menstrual period seems to affect them sometimes in a similar manner; the tumor has often been observed to increase perceptibly during the time of the menstrual discharge, and to diminish again after its cessation. The diagnosis of fibrous and cellulo-fibrous tumors must mainly be deduced from their history and their consistence. Their relatively very slow development, their comparative hardness, their uniform consistence, the absence of pain, and the normal state of the integuments covering them, serve to distinguish them from cysts or from cancerous tumors. Sometimes, however, the diagnosis may be rendered more difficult, owing to the suppuration or mortification which is liable to occur in growths of very large size, and the true nature of the tumor can only be ascertained by microscopic examination.

Fibrous tumors of the labia are rarely amenable to local or constitutional remedies. In the early stage of their development, a mild course of mercury may be tried, and alternated with the use of iodide of potassium, also an agent of some consequence in the softer forms of more recent fibrous growths; frictions with ointments containing mercury, iodine, or bromine, and stimulating embrocations or fomentations may be ordered. But the surest remedy is extirpation, which should always be advised when the tumor becomes a source of annoyance to the patient. The operation is generally of easy execution, and, owing to the loose condition of the cellular tissue of the labia in which the tumor is imbedded, it can, in most cases, be readily removed by enucleation. Some surgeons have advised total amputation of the diseased labium, but this dangerous procedure could be justified only in cases where the nature of the tumor is suspicious, or where the labium has undergone excessive elongation. In the latter case an embarrassing length of the flap can, however, always be avoided by including a sufficiently large piece of skin in the elliptical incision, with which the extirpation is usually commenced. It will be well to remember, in this connection, that the skin of the labium, even after very great enlargement, is still capable of considerable retraction. More recently, tumors of the labia have been removed by means of the écraseur. M. L. Paravicini reports a case in the *Ann. Univ. Agosto e Sett.* 1858, in which he used this instrument successfully for the extirpation of an atheromatous cyst of the size of a hen's egg, which occupied the lower two-thirds of the labium.

*Encysted tumors* are occasionally met with in the labia. They attain various sizes, and may even become as large as an infant's head. They are circumscribed, more or less fluctuating, firmer than œdema, but not as hard as fibrous tumors. Churchill describes them as being semi-transparent. This is by no means always the case, as the nature of their contents is very variable, and they are covered with more or less cellular tissue when they are deep seated. The skin covering them is rarely changed in color; in some cases, however, it has been found of a bluish or of redish-brown hue. Encysted tumors consist generally of but one cavity, which is filled with fluids of various character; the contents may be a serous, glairy, puriform or dark-colored liquid; sometimes they are more or less solid. The symptoms produced by encysted tumors of the labia are likewise referable solely to their situation and size; it is only when they are attended by inflammation they may give rise to more troublesome effects. Encysted tumors may be caused by a fall or blow on the soft parts a long time antecedent to their formation. Another species of cyst is owing to certain morbid alterations of the vulvo-vaginal gland of Bartholini, as described by *Huguier*, in his work, *Des maladies des follicules vulvaires et de la glande vulvo-vaginale.* These cysts are either owing to distention of the excretory canal and its ramifications, the orifice of which has become naturally or accidentally obliterated, or, which is more frequently the case, they are developed in the lobules of the gland, the follicles of which have ceased to communicate with the excretory duct, and have become more or less dilated. Owing to their development out of the glandular structure, these cysts assume sometimes curious forms, being ramified, and sending out processes. The vulvo-vaginal cysts occasion generally but little functional trouble. In some women, however, the cyst may become turgid and congested during menstruation, and may attract the attention of the patient by its increased volume and tenderness until it regains its former indolence with the cessation of menstruation. In other cases the cysts become more painful and voluminous after excessive fatigue, or coitus; and if these causes continue to operate, they may give rise to inflammation in the cysts and the surrounding cellular tissue, and produce an abscess of them and the vulvo-vaginal gland. The diagnosis of encysted tumors cannot always be made out with accuracy, although in the majority of cases their benign character is sufficiently apparent, and their consistency and the presence of fluctuation serve to distinguish them from fibrous tumors. Cysts of the labia are sometimes liable to be mistaken for inguino-labial or pudendal hernia, as described by Sir A. Cooper. Either of these tumors may occur in the same situation, and their touch is equally soft and elastic; on more accurate investigation, however, the distinct symptoms of the more serious affection of the two are found to be absent, for the cyst does not swell or become enlarged on coughing, and the swelling cannot be traced upward into the abdominal ring, or into the cavity of the pelvis. Mr. Ashwell mentions a case in which the diagnosis remained uncertain, until an explorative puncture was made, and the escape of some sanguinolent mucus removed all doubt. Encysted tumors of the labia can seldom be cured by any other than by surgical treatment. When the tumor is large the most certain plan is to dissect out the entire cyst, care being taken not to leave the slightest remnant, otherwise reproduction will be certain to occur. If the tumor is superficial and of smaller size, or if in consequence of the exudation around it, it adheres so intimately to the surrounding cellular tissue that enucleation must be renounced, the cyst may be opened by a large incision, its contents evacuated, and the cavity filled with lint smeared with an irritating ointment, or cauterized with nitrate of silver, or acid nitrate of mercury. Other eligible modes of treatment are to insert a seton through the tumor so as to produce suppuration, or to employ iodine injections in the same manner as in hydrocele of the vaginal tunic of the testicle.

*Fatty tumors* are not rarely developed in the parenchyma of the labia, especially in the vicinity of the mons veneris, and may rapidly grow to a considerable size. In other respects they have all the characteristics of fatty tumors in other parts of the body, and require the same mode of treatment.

*Scirrhous and cancerous tumors* may occur sometimes as a primary affection in the labia, but have more frequently their seat in the nymphæ or the clitoris; at other times they are but secondary and accompany analogous affections of the uterus and vagina. Cancer of the vulva presents the same character and symptoms as that of the uterus and vagina, and requires essentially the same treatment, but permits, on account of its situation, a more energetic employment of local measures than the former.

II. *Cases of Fistule of the Anus; Operation; Cure.*—1. Alexander

B., twenty-six years of age, a laborer, was admitted into the surgical wards of the Philadelphia Hospital, in January, 1860. The patient is a man of robust constitution, strong muscular development, and florid complexion. He is intemperate in his habits, and suffers from habitual costiveness; his health is in other respects generally good. About six years ago he noticed a small, hard swelling at the left side of the anus, which gave rise to much pain, especially during defecation; for several days the swelling increased in size, and finally burst, giving him immediately great relief. The abscess did not close up, and has been discharging ever since an offensive purulent matter, mixed sometimes with blood. On examining the patient, Professor Gross found the opening of the fistule at the distance of about eight lines from the anus, on its left side. A probe passed into the orifice penetrated to the depth of about one inch and three-quarters, and was found to emerge from an opening in the mucous membrane of the bowel, situated about four lines above the verge of the anus. The skin around the external opening was discolored and tender.

*Operation.*—The patient was put on low diet; a dose of castor-oil was administered the day previous to the operation, and the bowel was washed out with tepid water. A grooved director was inserted through the fistulous passage into the rectum, and its extremity brought out at the anus, and placed against the opposite buttock. A curved bistoury was then passed along the groove of the director, so as to divide rapidly the superimposed structures.

After the operation a thin tent, well oiled, was inserted into the wound, one end of it being passed some distance up the bowel to insure its retention. A compress was applied over the parts, and the whole retained by a T-bandage. The patient having been put to bed, a grain of morphia was administered to allay pain and to keep the bowels at rest for the succeeding forty-eight hours. On the third day a dose of castor-oil was administered. Under the use of the water-dressing, the wound filled up readily with healthy granulations, and is now, about two weeks after the operation, nearly cicatrized.

2. Francis K., fifty-four years of age, a carpet-weaver, was admitted into the surgical clinic of the hospital, in January, 1860. The patient is of thin, slender form; he is said to be temperate in his habits, and has been commonly in good health. His bowels act with regularity, and he has not suffered from hemorrhoids. The patient complaining of a slight cough, his lungs were examined, but no evidence of tubercular disease could be detected. About seven years ago he suffered from an abscess on the right side of the anus, the formation of which he ascribed to having been exposed to cold and wet. The swelling soon burst, and during the whole period which has elapsed since, there has been almost constantly a discharge of thin pus, and sometimes, when the bowels were in a relaxed state, of fecal matter or of gas, through the opening. On examination, the parts around the anus were found to be indurated and discolored; about one inch to the right of the anus an opening existed, surrounded by a small papillary mass of granulations; a probe introduced into it passed to a distance of about two inches and a quarter. On inserting the left index finger into the rectum the point of the probe was felt protruding through an internal opening situated about half an inch above the verge of the anus.

The patient having been put under preparatory treatment, the fistule was completely laid open in the same manner as in the first case. The after-treatment was also conducted upon similar principles. The sore healed up slowly but steadily, and the cure is now, three weeks after the operation, nearly complete.

*Remarks.*—In presenting the two cases reported above to his class, Professor Gross made the following remarks on the disease of which they were an illustration:—

By fistule of the anus is meant an abnormal track extending from the rectum to the skin. The immediate cause of the complaint is an abscess formed in the loose cellulo-adipose tissue around the lower part of the rectum, which bursts externally near the anus, and leaves an opening for the discharge of matter indisposed to heal. The lower bowel having been more or less extensively detached from the surrounding structures during the suppurative process, a sinus is formed in this way, which burrows close to the outer surface of the mucous membrane of the rectum; the latter, constituting but a thin barrier between the sinus and the interior of the bowel, finally is perforated by ulcerative action, and thus is formed a passage which admits, in many cases, of the escape of gas, mucus, and fecal matter from the interior of the rectum to the surface of the skin. When a communication of this kind exists, the case is called one of *complete* fistule. If, on the other hand, the wall of the bowel retains its integrity, the abnormal track extending merely from the skin to its outer surface, then the fistule is incomplete, and is called, in reference to its situation, an *external blind fistule.* Professor Gross believes that a fistule of this kind is much more rare than is commonly assumed, and that in many cases, supposed to be of this nature, the surgeon, either from want of dexterity or other causes, is unable to detect an opening in the rectum, when one is actually present. This view receives support from the opinion of so high an authority as Sir B. Brodie, who even stated, in a valuable lecture on this subject, (*Lancet*, 1843-4, vol. i.,) that he is satisfied that the inner opening always exists. Still rarer than the external blind fistule is what is described in books as *internal blind fistule,*

that is, a cul-de-sac extending from the rectum or ano-rectal cavity into the subjacent cellular substance without perforating the common integument. In many cases apparently of this nature, there may exist originally a complete fistule the external opening of which has closed for a short time, the spot being indicated by redness and induration; but sooner or later it reopens, and the discharge returns, or a fresh opening is formed at some distance off. It may happen, however, that the original ulcerated opening in the rectum being large, the matter from the abscess formed in the cellular tissue outside finds its way so readily into the bowel that the abscess does not burrow toward the surface. Cases of this kind are of very rare occurrence.

*Anatomy of fistule.*—Although fistule of the anus is a very common disease, and apparently very simple in its nature, still there are some points in the anatomy of the disease which require the particular attention of the surgeon, and without a complete knowledge of which he will be unable to conduct the treatment successfully. The points to be considered in this connection are the internal orifice of the fistule, its external orifice, and its course.

In the great majority of cases there exists but one *internal orifice;* in rare instances, however, several of them may be present. In the case of a gentleman, twenty-four years old, of Ripley, Mississippi, whom Professor Gross treated for this disease in 1855, there were four distinct internal openings, about half an inch above the verge of the anus, two on the left side, one in front, and one on the right side. It is of high importance, in regard to the successful management of a case of fistule, to ascertain whether the internal opening is single or multiple; but this information is by no means always easily gained. The presence of several internal openings may be suspected, especially when the abscess has been large, when the rectum has been separated to a considerable height from the surrounding tissues, and when the opening of the abscess has been deferred to a very late period. In regard to the situation of the internal orifice very erroneous opinions have been entertained until a comparatively recent period; it was generally searched for as high up as two, three, or even four inches from the margin of the anus, and the patient was consequently subjected to a rather dangerous and difficult operation. The observations of Sabatier, Larrey, and Ribes, which have been confirmed by those of Chelius and Sir B. Brodie, have, however, clearly shown that the inner opening, in the large majority of cases, is situated immediately above the junction of the mucous membrane and the common integument, or rarely higher up than three, four, or five lines from the verge of the anus. In searching for the internal orifice, the probe must therefore be directed toward the lower end of the rectum. Occasionally the orifice is a little higher up, and in a few rare instances it has been known to be ten, fifteen, or even eighteen lines above the verge of the anus; but examples of this kind are exceedingly rare. The shape of the internal orifice is circular or ovoidal; its size rarely exceeds a line in diameter.

The *external orifice* is subject to a greater diversity, in regard to number and situation, than the internal orifice. Though it is more frequently single, instances are not rare in which a number of external openings exist; sometimes these are situated only a few lines from each other; at other times, at a distance of several inches, although they may correspond with but a single internal orifice. Professor Gross has a specimen in his private collection in which there are as many as seven external openings, giving the cutaneous surface quite a cribriform aspect. The external opening is generally situated at the side of the anus, at a distance varying from half an inch to several inches. Occasionally it is found in front toward the scrotum, and sometimes, though rarely, directly over the extremity of the coccyx. Its shape is usually very irregular, and its size varies in different cases, from that of a pin-head to that of half a dime. Its site is generally indicated by a small mammillated mass of granulations, or by a small reddish point, perhaps not larger than a flea-bite, and quite tender to the touch.

The fistulous *track* which is left after the abscess has burst and its walls have contracted, varies considerably in regard to its situation and direction. Most commonly it is situated at the side of the rectum, but sometimes it lies in front of it, or near its posterior wall. Experience has shown that the wound made by the operation generally heals more readily when the fistule is situated at the side of the anus, than when it corresponds with the median line. This difference is explainable by the anatomical disposition of the parts. The fistulous track forms with the rectum an angle which is the more acute the nearer the external opening is to the anus, and the higher up the internal orifice is situated; occasionally, however, a fistulous passage is met with which is nearly horizontal in its direction, and which opens into the rectum at a right angle. The fistulous track may be either straight, curved, or tortuous; in some cases it may even form an angle in one part of its course, so that the introduction of a probe into its whole length is rendered difficult, or even impossible. In order to pass a probe, under such circumstances, into that part of the fistule which is next to the rectum, it may become necessary first to divide the side of the angle which terminates in the external orifice. The length, size, and shape of the fistulous track are just as variable as its direction. While in some instances it is but a few lines long, and hardly large enough to admit the finest probe, in others it is several inches in length, and so capacious as to permit the passage of the largest instrument. Its shape may be cylindrical, slit-like, or flattened. Instead of a single track, two, three, or more may be present; they generally communicate with each other laterally, although they may all concentrate at one internal orifice. An illustration of this arrangement is found in the *horseshoe fistule* described by Curling, in which there is an external orifice, at each side of the anus, leading to fistulous passages which pass to the back of the rectum and communicate with the gut at this part by a single orifice, so as to give the fistule the shape of a horseshoe. Experience seems to show that the fistulous tracks are most numerous and most extensive when the fistule is associated with stricture of the bowel. In external blind fistule the track terminates above in a pouch or hollow, which is sometimes quite capacious from the great destruction of the cellulo-adipose tissue around the gut. Soon after its formation, the fistulous track is lined by a layer of lymph which, in time, is transformed into a smooth, adventitious membrane, closely assimilating itself to the mucous membrane of the bowel. When the disease is of long standing, the sides of the track are often dense and callous.

The *discharge* of the fistule is generally a thin, purulent fluid. It is more or less copious in different cases, and varies at different times, sometimes becoming so thin and scanty that the patient thinks himself near a cure, until a fresh irritation is set up, and the complaint becomes as troublesome as before. In complete fistule the track gives, occasionally, vent to flatus, and to fecal matter.

*Condition of the parts in the neighborhood of the fistule.*—In the different kinds of fistule which may form in its vicinity, or communicate with its interior, the rectum is commonly more or less separated from the surrounding tissues. Sometimes it is denuded, but to a trifling extent; at other times the separation implicates a large part of the circumference of the gut, and extends so high up that even that section of the rectum contained in the small pelvis may be completely isolated. Such extensive separations are always serious in their consequences, and may be even fatal. It is worthy of notice, that the inner orifice of the fistule is situated, most frequently, not at the highest point of the denuded part, but a considerable distance below it, and that, in order to establish a cure, it is not necessary to carry the knife higher up than that orifice. The cellular tissue and skin for a considerable distance around the fistule are generally more or less indurated, hypertrophied, discolored, and tender in consequence of chronic inflammation, which is kept up particularly by the passage and retention of mucus and feces in the parts more immediately implicated in the disease. The skin is sometimes undermined to a large extent. Small abscesses are developed, discharging thin, unhealthy matter. The sac formed by the loss of the cellular tissue around the fundament becomes the receptacle of pus and fecal matter, giving thus rise to fresh annoyances, attended with fetor and inflammation, and leading to new abscesses and additional passages; defecation is painful, the bladder is irritable, and the patient finds it difficult to sit, or to ride on horseback, and is unable to walk much. Even when the seat of the disease is free from inflammation and tenderness, he is annoyed by a constant discharge, which stains the linen and keeps the parts uncomfortably moist.

The *general health* of the patient afflicted with anal fistule deserves much attention; sometimes it is not impaired to any perceptible degree; in other cases, however, there is considerable emaciation, with gradual loss of strength, and the suppuration is very abundant, and accompanied by fever; all these symptoms may be caused by the local disease, there being no serious lesion of any of the internal organs present. Under these circumstances, the operation is plainly indicated. It is different, however, when the emaciation, the fever, and all the other symptoms are the consequence of some organic disease, and the fistule is merely a coincident affection, as, for instance, in phthisis. Experience has shown that in cases of this kind the operation hastens the progress of the internal disease, and expedites the fatal termination. Believing the establishment of a fistule in consumptive persons an effort of nature to stay the morbid process going on in the lungs by drawing off the irritation from these organs, Heurteloup has even proposed to make an artificial fistule in cases of pulmonary phthisis, by means of instruments invented by him for this purpose. His plan of treatment has found, however, no imitation, as it is useless, and subjects the patient to unnecessary annoyance.

*Diagnosis.*—The diagnosis of anal fistule is founded upon the history of the case, the course and nature of the preceding abscess, the absence of those symptoms which are proper to perineal fistules and fistules resulting from caries of the bones of the pelvis or spine, and finally upon the symptoms peculiar to the disease. They are the following: If the fistule is complete it gives occasionally vent to gas, and the purulent or sanguinolent discharge from it is often tinged with fecal matter; a probe, cautiously introduced into the fistule, will come in immediate contact with the finger inserted into the rectum; when the fistule is so tortuous or angular that the sounding of it is difficult and unsatisfactory in its results, an injection of milk or some colored fluid may be made into it, a part of which will come back through the anus. The external blind fistule is distinguished from the complete by the absence of the symptoms which indicate the perforation of the rectum. When the fistule is an internal blind one, the patient discharges pus through the anus; in the neighborhood of the latter an indurated place will be found, in the middle of which there is often a red, soft, compressible, sometimes prominent, at other times depressed part; on pressure upon this spot, a small discharge of matter is frequently effected by the anus, and sometimes the expulsion of air from the cavity of the abscess into that of the intestine may be distinctly felt, or clearly heard; the stools, particularly if hard and requiring force for their expulsion, are sometimes smeared with matter; the patient is rarely perfectly free from a dull kind of uneasiness, especially if he sits for any considerable length of time in one posture; on defecation the swelled parts become occasionally the seat of heat and pain. On introducing the finger into the rectum, the internal orifice may sometimes be detected.

The *examination* of the parts in a case of anal fistule should be conducted with great caution and gentleness. When they are much inflamed and tender, they should be brought into proper condition for the examination, by rest, leeches, iodine, astringents, and anodyne enemata. The morning previously the bowel should be washed out well with tepid water. The best instrument to conduct the exploration is a common pocket-probe. The patient may be examined lying on the side, or stooping over a table or chair, or, better still, resting on his knees and forearms. The probe is inserted, well oiled, through the external orifice, which is, as previously stated, generally situated in the middle of a small papilla on the skin. The left index finger, being in the rectum, feels for the point of the probe, and thus assists in guiding it to the internal orifice of the fistule, provided this is present, or against the side of the gut, when the fistule terminates in a cul-de-sac. In passing the instrument, it is best to give it a considerable inclination toward the anus, the usual situation of the internal orifice being just within the sphincter. The manipulation should be performed without the use of the slightest force, as the cellular tissue around the rectum is so loose and yielding that, without care, a passage may be made where none existed previously. If much difficulty is experienced in detecting the internal orifice, use may be made of the speculum, and, if necessary, of an injection of milk into the external orifice, as mentioned before; the appearance of the fluid at some spot on the mucous membrane will indicate the situation of the internal orifice.

*Etiology.*—The causes of abscess, and consequently of fistule of the anus, are very various. Habitual costiveness and hemorrhoids have a great influence in the production of the disease. Sometimes an external injury, or the lodgment of some foreign body, which may penetrate through the mucous membrane of the bowel and sphincter muscle into the cellular tissue, may give rise to inflammation, abscess, and fistule. The disposition of the pouches of the rectum, which are wide and deep, and open from below upward, as well as the resistance of its sphincters, serve to detain hard, pointed, or irregular bodies which have been swallowed, such as a fish-bone, a pin, a kernel, etc. In some cases ulceration of the rectum, originating from chronic dysentery, syphilitic disease, or tubercular deposits, may cause perforation of the bowel, and consequently abscess and fistule; but such cases are very rare, and in most instances the perforation is the secondary lesion, following upon inflammation and abscess of the neighboring tissues. It has long been a common belief in the profession that consumptive persons are particularly prone to the formation of fistule, but this doctrine is not borne out by facts. According to the statements of Andral and Louis, who have investigated this subject extensively, anal fistule is rarely met with in phthisis. Age seems to exercise some influence upon the formation of the disease. Occurring at all periods, it is most common in middle-aged persons, childhood and old age being almost exempt from it. Professor Gross has, however, witnessed it several times in young children, and in one case in a girl only three years and a half old. Recently he treated a boy, six years old, at the clinic of the Jefferson Medical College, in whom the disease began at the age of nine months. After the age of fifty it is very rare, while after that of sixty it is almost unknown, which, probably, is owing, in a great measure, to the relaxed condition of the rectum and sphincter in old people, rendering the mucous membrane less liable to irritation. The disease is more frequent in men than in women, but in what ratio has not been ascertained. Judging from his own experience, Professor Gross is inclined to look upon the difference as very marked, for, while he has seen a large number of cases in the male, he has met with very few in the female. His practice having always been pretty equally divided between the two sexes, he is sure that the difference could not be accidental. The cause for this unequal distribution of cases among the two sexes is unknown. The influence of occupation is not marked, as the disease occurs in all classes of persons—in laborers, farmers, mechanics, merchants, and professional men. It is generally assumed that individuals who are in the constant habit of standing, or riding on horseback, are particularly liable to the disease; but if this be the fact, the experience of Professor Gross does not go to substantiate it.

In considering the immediate cause of fistule, the question arises why an abscess in the neighborhood of the anus does not close spontaneously after the discharge of its contents, but leaves a fistulous sore. The anatomical disposition and function of the parts serve to explain this circumstance. If it is considered that, from the looseness of the skin, the action of the elevator and sphincter muscles, and the movements of defecation, the diseased parts are subjected to constant disturbance, the persistence of a sinus after purulent formations is not at all astonishing; and even without this interference, the occasional escape of a little feculent matter into the passage, in cases where the fistule communicates with the interior of the rectum, would be amply sufficient to prevent the closure of the parts. The tendency of abscess of the anus to the formation of fistule can be counteracted only by an early and prompt treatment of the primary affection.

*Prognosis.*—From what has been stated above, it may be presumed that fistule of the anus rarely heals spontaneously; indeed, the disease may continue for an indefinite period, unless cured by operative interference. Anal fistule was formerly considered a dangerous and unmanageable disease; but this opinion is no longer entertained, since the anatomy of the disease and the causes which keep it up are more accurately understood, and a simple as well as rational mode of treatment has been adopted. It is hardly necessary to mention that the prognosis is subject to modifications which depend upon the varieties of the disease and the different circumstances under which it occurs.

*Treatment.*—Different methods of treatment have been proposed for the cure of fistule of the anus, such as compression, irritating injections, cauterization, excision, the seton, and division; the last two operations are alone practiced at the present time by professed surgeons; the other methods have been rejected as useless, and even obnoxious, and are only occasionally adopted by ignorant empirics or charlatans. The most efficient operation consists in the division of the parts intervening between the inner and outer orifices, including the fibres of the external sphincter. Professor Gross considers the following the most eligible mode of operating: A grooved director is inserted into the abnormal track, its extremity brought out at the anus, and placed against the opposite buttock, when the superimposed structures are divided with the knife, passed along the groove of the director. In this manner the operation may be performed in a few seconds, and without the slightest risk of doing injury to the rest of the bowel. When the fistule is very short, Professor Gross sometimes carries a narrow probe-pointed bistoury through it, and effects its division in that way, counter-pressure being made against the extremity of the instrument by the finger in the rectum. When there is no internal orifice, as when the fistule is a blind external one, an opening must be made into the bowel at the usual height with a sharp-pointed director, when the parts are divided as in the former case. In those rare cases in which the fistule is a blind internal one, the sac in which it terminates below must be laid open with the knife; the fistule is thus rendered complete, and may be treated in the usual manner. When the track is of long standing, and incrusted with organized matter, the cure will be rendered more certain, as well as more rapid, by notching its bottom, or scraping away the adventitious matter. In those cases where several external openings exist, leading to different sinuses communicating with each other, each of them must be divided, so as to make one cavity of the whole. If there are two internal orifices on the same side, situated at the usual height, they should, if possible, be included in one incision; if this is impracticable, the upper one may be divided in the usual manner, and the lower one be laid open into it. Should the upper orifice, however, be situated high up, it is advisable to operate only upon the lower one, trusting that the upper one and its sinus will close after the sphincter has been rendered inactive. A cure can, however, not be relied upon under these circumstances; and as a long incision into the rectum is dangerous, on account of hemorrhage, it will be better to use the seton in cases of this description. When there is a greater number of internal openings, situated at different parts of the bowel, the employment of the knife would hardly fail to be followed by loss of power in the sphincter muscle; the treatment by the seton is then indicated.

The loss of blood, resulting from the operation described above, is but slight, and rarely amounts to more than a few drachms; whereas, in the old process, where the knife was carried several inches above the anus, the hemorrhage was quite profuse and even fatal. Considerable bleeding may be, however, occasionally observed in cases of long standing, where great induration of the parts exists, and the vessels are unable to retract into the condensed cellular tissue. The hemorrhage can, under these circumstances, generally be controlled without difficulty by exposure of the wound to cold air, and the application of pounded ice, astringent lotions, or compression.

The treatment after the operation consists in inserting a slender slip of oiled lint, one end of which is to be passed up several inches at the opposite side of the bowel, and the other end laid across into the wound. A compress and T-bandage are then applied, and the patient is put to bed. The bowels, which should have been made to act freely before the operation, are not to be disturbed for two or three days afterwards, and for this purpose a full dose of morphia or opium should be administered. At the end of this time a mild laxative or enema may be prescribed. The dressing after this period should be renewed every day. The wound commonly closes readily by granulation, and the functions of the sphincter are unimpaired.

The operation by the *seton* for the cure of fistule was originally proposed by Hippocrates and Celsus. Having been out of use for a long time, Foubert brought it again before the notice of the profession, and Desault invented instruments to apply it in cases where the internal orifice is situated very high up. The substance employed by Professor Gross for making a seton is silk twist, about the thickness of common twine; he inserts it by means of a silver probe, very slender, and about two inches and a half long. The ends of the ligature are secured over a small button, having two holes at opposite points, and are tightened once every second or third day until it cuts its way out, as it usually will in less than a fortnight. The advantage of this treatment is, that it is almost free from pain and does not interfere with the occupation of the patient, as he is able to walk about, having merely to pay attention to cleanliness, to his diet, and the state of his bowels. For reasons already stated, the seton is preferable to the knife in cases where the internal orifice is located unusually high up, or when there is a greater number of internal orifices situated at different parts of the bowel. In a case of the latter description, previously adverted to, Professor Gross inserted not less than three setons, and had the satisfaction of effecting a complete cure in less than a month. The seton may find its application also in cases complicated with serious organic disease of the lungs, heart, or liver, where the use of the knife is contra-indicated on account of the danger which would attend the sudden arrest of a habitual discharge. In the majority of cases, however, it will be the safest plan either not to interfere at all with a fistule occurring in a consumptive person, for fear of hastening the progress of the internal affection, or, if an operation is indispensable on account of the local suffering, to establish an issue upon the chest.

If an operation for this or other reasons must be renounced, the patient may be rendered comparatively comfortable by attention to his diet and bowels, and the observance of cleanliness.

III. *A Case of Scirrhus of the Mammary Gland; Suppuration and Sloughing of the Tumor, and Final Cicatrization.**—Margaret Sweeny, fifty years of age, a cook, unmarried, of dark complexion, and middle stature, was admitted into the surgical wards of the Philadelphia Hospital, in November, 1859. Previous to the time when her breast became affected her health had been good, and her menstrual function had been performed regularly until about four years ago, when it ceased.

---

* In obtaining the history of this and other cases, we are indebted to the kind assistance of Drs. B. S. Wood, Lodge, and Goodman, Resident Surgeons of the Philadelphia Hospital.

Her parents had died of old age. About eight years ago she discovered a small hard lump, of the size of a bullet, in her right breast, some distance above the nipple. The tumor was occasionally the seat of sharp, shooting pain, which, however, was so slight that she did not pay much attention to it, and continued at her occupation. For five years the enlargement of the swelling advanced very gradually. About three years ago, when the tumor had attained the size of a walnut, the skin covering it became discolored and red, the swelling rapidly increased, and became the seat of violent pain, which disturbed her rest at night; for several days these symptoms augmented in severity, and the tumor finally burst. The patient dressed the parts with a bread and milk poultice. After the discharge had continued for five weeks it ceased, the opening closed, and the patient followed her occupation as usual. From that time the same symptoms recurred at intervals of about four or five months, and were each time followed by a discharge lasting several weeks. At the beginning of October, 1859, the tumor, which was about as large as a hen's egg, became the seat of insufferable pain; the affected breast was seized with violent inflammation, and soon swelled to twice its natural size; in the middle of October the skin over the tumor burst, and a copious and fetid discharge ensued. On the nineteenth of November, when the patient was admitted into the hospital, the skin, including the nipple of the affected gland, was found to be destroyed extensively by ulceration; the sore had a foul, unhealthy appearance, and was the seat of sloughing; the discharge from it was of a sanious nature and extremely fetid; the affected breast was hard, inflamed, exquisitely tender, and the seat of a violent pain, which was increased on the slightest movement of the arm; the patient suffered from high fever, and was very much emaciated.

The diseased parts were dressed with an emollient poultice sprinkled with powdered opium, and the patient was put on the use of tonics alternated with purgatives. The process of sloughing went on rapidly; on the sixth day, the greater part of the affected tissues had separated; a lotion containing one drachm of acid nitrate of mercury in six ounces of water was substituted for the poultice. On the twelfth day, after the admission of the patient, healthy granulations appeared at the bottom of the cavity left after the detachment of the sloughs. The parts were dressed with opiate cerate, under the use of which cicatrization finally took place. About a month after the commencement of the treatment the parts were completely healed.

In January, 1860, when Professor Gross exhibited the case to his class, the patient was apparently in very good health; she had gained much in flesh, and her complexion did not show any of the peculiarities commonly ascribed to cancerous cachexia. The left mammary gland had been completely destroyed; its former situation was indicated by a cicatrice four inches long and two inches wide, red, and highly vascular, having inverted edges, and looking very much as if produced by a burn. The glands in the axilla were but little enlarged. But within the last few weeks a number of small tubercular elevations, evidently of a scirrhous nature, made their appearance at the edges of the cicatrice, which were occasionally the seat of lancinating pain, and also in the integuments around.

In making some remarks on the very rare occurrence of cases where nature makes an effort at the extrusion of a cancerous tumor, Professor Gross stated that he had seen but one other instance of it. The patient was an elderly lady, fat, and otherwise healthy, who had a medium-sized tumor in one of the mammary glands which had troubled her for several years. All of a sudden, without any assignable cause, inflammation set in, and in a few weeks the whole mass was lifted from its bed as neatly as if the operation had been performed with the knife, the cavity being afterwards filled up with healthy granulations. Some time subsequently, the disease broke out in the axillary lymphatic ganglions, and made rapid strides toward a fatal termination.

IV. *A Case of Neuralgia of the Stump; Cure.*—Jacob B., twenty-eight years old, of light complexion and strumous habit, was admitted into the surgical wards of the Philadelphia Hospital, in May, 1859, on account of extensive scrofulous disease of the ankle-joint. Amputation being deemed necessary, the limb was removed at its lower third by the flap operation. A few days after the operation, the parts over the tibia commenced to slough, and the bone finally protruded. After the removal of a triangular piece of bone, about one inch in length, the stump healed without difficulty, and gave no further trouble. In January, 1860, when the case came under the observation of Professor Gross, the patient commenced to complain of a sharp, lancinating pain over the end of the tibia, which was worse at night, and was increased on pressure. He had no fever, nor was his general health disturbed in any other way. He was ordered to take ten grains of sulphate of quinia and half a grain of sulphate of morphia, at night, and the stump was well protected from cold, friction, and pressure. The patient recovered within two weeks.

*Remarks.*—On presenting this case of neuralgia of the stump to his class, Professor Gross made the following remarks: A disagreeable, frequently indeed a most distressing, secondary affection which may follow amputation, is neuralgia. It comes on at a variable period after its performance, and often continues, despite the most judicious and persevering efforts at relief, to molest the patient during the remainder of his life. Supervening generally without any assignable cause, it is usually most common in nervous, irritable persons, who are subject to the disease in other parts of the body. Females are more prone to it than men, and in them the attack frequently coincides with the eruption of the menses. Sometimes the disease is periodical, especially in residents of malarious regions, the paroxysms coming and going very much as in intermittent fever. Most commonly, however, the pain is omnipresent, one portion of the day being as liable to bring it on as another. It is generally of a darting, shooting nature, or dull, heavy, and aching, and is invariably aggravated by damp states of the atmosphere, fatigue, and disorder of the digestive apparatus. In the more violent forms of this affection, the immediate cause of the difficulty is a bulbous enlargement of the nerves ramifying through the stump. This degeneration takes place, to a greater or less extent, after nearly every amputation, and is therefore to be considered as a disease only when it exists in excess. Under such circumstances, the tumor, which sometimes attains the size of a hickory-nut, or even of a pullet's egg, is of a firm, dense consistence, and is composed of a strong, fibrous stroma, inlaid with hypertrophied and curiously interlaced nervous trunks and filaments. It is, in fact, a true *neuroma*. The accompanying pain is exquisite, and the part is so sensitive as to be intolerant of the slightest touch; the general health is much affected, and the patient is remarkably susceptible of atmospheric vicissitudes, every change in the weather, from warm to cold and dry to wet, being followed by an increase of suffering. This malady is of a much more serious character than the other, and requires proportionably stronger measures. In general, nothing short of removal will avail: by excision, if the tumor be single and easily accessible; by amputation, if it be multiple and deep seated. For the milder varieties of neuralgia the ordinary remedies will sometimes suffice, the same as in neuralgia in other parts of the body, especially quinine, or, if the patient be anæmic, quinine and iron, combined, in either case, with strychnine and arsenious acid, belladonna, stramonium, or aconite; the effects of the articles being studiously watched, lest an over-dose be given and life placed in jeopardy. Sometimes good effects accrue from the exhibition, every night, of colchicum and morphia, administered in full doses at bedtime, as one drachm of the wine to a grain of the salt. The remedy is particularly valuable in subjects of a rheumatic state of the system. Locally, iodine, blisters, issues, and other counter-irritants may be used along with anodyne embrocations and injections. Particular attention should be paid to the protection of the stump from cold, friction, and pressure.

